# Function and energy consumption constrain neuronal biophysics in a canonical computation: Coincidence detection

**DOI:** 10.1371/journal.pcbi.1006612

**Published:** 2018-12-06

**Authors:** Michiel W. H. Remme, John Rinzel, Susanne Schreiber

**Affiliations:** 1 Institute for Theoretical Biology, Department of Biology, Humboldt-Universität zu Berlin, Berlin, Germany; 2 Center for Neural Science, New York University, New York, New York, United States of America; 3 Courant Institute of Mathematical Sciences, New York University, New York, New York, United States of America; University of Pittsburgh, UNITED STATES

## Abstract

Neural morphology and membrane properties vary greatly between cell types in the nervous system. The computations and local circuit connectivity that neurons support are thought to be the key factors constraining the cells’ biophysical properties. Nevertheless, additional constraints can be expected to further shape neuronal design. Here, we focus on a particularly energy-intense system (as indicated by metabolic markers): principal neurons in the medial superior olive (MSO) nucleus of the auditory brainstem. Based on a modeling approach, we show that a trade-off between the level of performance of a functionally relevant computation and energy consumption predicts optimal ranges for cell morphology and membrane properties. The biophysical parameters appear most strongly constrained by functional needs, while energy use is minimized as long as function can be maintained. The key factors that determine model performance and energy consumption are 1) the saturation of the synaptic conductance input and 2) the temporal resolution of the postsynaptic signals as they reach the soma, which is largely determined by active membrane properties. MSO cells seem to operate close to pareto optimality, i.e., the trade-off boundary between performance and energy consumption that is formed by the set of optimal models. Good performance for drastically lower costs could in theory be achieved by small neurons without dendrites, as seen in the avian auditory system, pointing to additional constraints for mammalian MSO cells, including their circuit connectivity.

## Author summary

Cellular design varies widely across neurons. Evolution is thought to have adapted the biophysical properties of each cell type to better support their specific function. The most influential among these neuronal parameters include ion channels and morphology. At the same time, we know that the brain is disproportionately expensive: it consumes ~20% of resting oxygen but only contributes ~2% to body mass. While both function and energy seem important constraints in neuronal design, it is unclear how neural systems simultaneously account for both factors and how potential tradeoffs between the two were decided in evolution. To explore this relation, we focus on a cell type with well-characterized function: MSO neurons of the mammalian auditory brainstem, which detect precisely coincident signals to encode sound source direction. Measurements of metabolic markers indicated that these cells are particularly expensive. Hence, they are exceptionally interesting candidates to better understand potential compromises between function and energetic demand. We use models of conductance-based neurons constrained by experimentally observed characteristics with parameters varied within a physiologically realistic range. Our study shows that neuronal design of MSO cells does not compromise on function, but favors energetically less costly cell properties where possible without interfering with function.

## Introduction

Morphology and membrane properties of neuron types in the brain show a large diversity. The systematic differences between cell types are assumed to be matched to the computations carried out by neurons and to support local network connectivity [1–3]. On the other hand, other constraints may shape neuronal design further. One such factor that has been discussed extensively over the past decades is energy efficiency [4,5]. The brain accounts for a disproportionately large part (~20%) of the energy budget [6], with metabolic energy being spent mostly on electrical signaling: synaptic input, action potentials and resting potentials [4,7,8]. These signaling costs arise primarily from the consumption of ATP by the sodium-potassium pump that maintains the sodium and potassium concentration gradients across the membrane [4]. Minimization of energy consumption has indeed been suggested as a constraint for cellular biophysics, e.g., for the properties of sodium and potassium currents underlying spike generation [9–13], cell morphology [14,15] but also for neural coding schemes [16–18]. It is, however, still not clear to what extent energy consumption competes with neural function in defining cell morphology and membrane properties.

**Fig 1:**
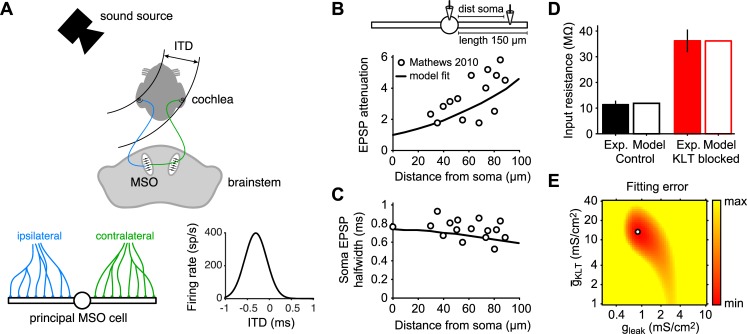
Processing of ITDs by MSO cells and fitting the membrane properties of a simplified MSO model to experimental data. (A) MSO cells use the time difference for sound to reach both ears (interaural time difference, ITD) to encode the location of a sound source in the horizontal plane. Top: Sound waves arrive at both cochlea and, via several intermediate stages, reach the principal neurons of the MSO nuclei in the brainstem. Bottom left: Fibers arriving from the ipsilateral (blue) and contralateral (green) ear are segregated on the two main dendrites of the principal MSO cells. Bottom right: MSO cell firing activity strongly depends on ITD because of precise coincidence detection of the ipsilateral and contralateral signals. (B) Schematic of minimal model of principal MSO cell. In experiments by Mathews et al. [25] an EPSC current waveform was injected in one dendrite at a distance of 0–100 μm from the soma (“dist soma”) and the voltage attenuation (i.e., the ratio of dendritic amplitude to somatic amplitude) was determined (circles). The experiment was simulated numerically with the default minimal model (solid curve; see Methods). (C) The width of the propagated EPSP at soma at halfmaximal amplitude as determined by experiments [25] (circles) and from numerical simulations of the default minimal model (solid curve). (D) Somatic input resistance determined in experiments [25] and from numerical simulations of the default model under control conditions (left) and with KLT blocked (right). (E) Densities for the leak conductance and KLT peak conductance were varied from 0.2 to 10 mS/cm^2^ and from 1 to 40 mS/cm^2^, respectively, and the sum squared error for EPSP attenuation, somatic EPSP halfwidth and input resistance was computed. The parameter combination with the lowest fitting error (open circle) was used for the default model. Panels B and C were adapted with permission from Fig 2 in ref. [25].

In this study we focus on a specific system that is known to have a high energetic demand: the medial superior olivary (MSO) nucleus of the mammalian brainstem [19,20]. We aim to understand the influence of energy consumption on the design of MSO principal neurons. To this end, we quantify the impact of crucial cellular parameters on the well-defined functional computation performed by these cells as well as their energy consumption. We can rely on the facts that 1) the function of the highly specialized MSO principal cells is well characterized and relies on the computation of the temporal coincidence between two inputs and 2) the cells’ metabolic demand is known to be particularly high. MSO principal cells play an important role in auditory perception, as they encode the direction of sound in the horizontal plane [21–23]. MSO cells accomplish this by exploiting the time difference for a sound wave to reach both ears: the interaural time difference ITD (Fig 1A). Sound waves are transduced into electrical signals in the cochleae of each ear and through several intermediate synapses reach the principal cells of the MSO nuclei in the brainstem. The excitatory inputs relaying the signals from the ipsilateral and contralateral ear each impinge on one of the two main dendrites of the bipolar MSO cells. The excitatory synaptic inputs can convey the ITD information to the MSO cells because they are phase-locked to the sound wave stimuli to each ear. The timing difference between the ipsi- and contralateral inputs is then used to encode source location through the fundamental neuronal computation of *coincidence detection* [1,24]. As a consequence, MSO cell activity varies strongly as a function of ITD, with different MSO cells having different ITDs for which they respond most strongly. MSO cells show sensitivity to ITDs in the range of tens of μs, meaning that at the behavioral level humans for example can discriminate changes of just 1–2 degrees in the angular location of a sound source [23]. To achieve this, MSO cells have specialized membrane properties, including a very fast membrane time constant (<1 ms) and a low-threshold potassium current, *I*_KLT_, both contributing to a very short input integration window [25]. Furthermore, MSO cell function is supported by the segregation of the ipsilateral and contralateral inputs to the two main dendrites [26]. The brain structures involved in auditory function, from the cochlear nucleus to the auditory cortex, are particularly expensive in terms of glucose utilization [19]. For MSO cells specifically, their computational function is associated with significant energy consumption because of the leaky membrane that underlies the fast membrane time constant and the very high input rates (hundreds of spikes/s) these cells have to process. Hence, MSO cells provide a valuable opportunity to investigate the constraints that both function and energy consumption create for neural properties.

In order to quantify and compare the sensitivity of MSO cells to ITDs and the associated metabolic cost, we here develop a minimal biophysical model of a principal MSO neuron that quantitatively matches previously reported experimental properties [25,27]. We then use this model to systematically explore how morphology and membrane parameters affect both the performance and the energy consumption associated with ongoing processing of synaptic input. We identify parameter ranges that combine good performance with low energy use and compare these with the experimentally-derived properties of MSO neurons. Overall, our results point towards a strong influence of functional performance on neuronal properties, that is combined with an energy-saving design, if achievable without compromises to function.

## Results

### Biophysical model of a principal MSO cell

To investigate how both the computational function and energy consumption of MSO cells constrain cell morphology and membrane properties, we systematically tested how the cell parameters affect performance and energy use. To this end, we developed a simplified biophysical model of a principal MSO cell based on anatomical and experimental data.

The bipolar morphology of principal MSO cells was captured by a soma compartment with two identical dendritic cables which, in agreement with anatomical data [27] were of constant diameter. Thus, three parameters describe the cell morphology: the surface area of the soma and the length and diameter of the dendritic cables. The membrane properties of MSO principal cells have been experimentally characterized by a very high membrane conductance and the presence of the low-threshold activated potassium current, *I*_KLT_ (see, e.g, ref. [25]). Both features were included in the biophysical model. For simplicity, we considered the KLT-current to have a uniform density throughout soma and dendrites. It was the only voltage-dependent current included in the model, the other membrane conductances that make up the high resting conductance of MSO cells (e.g., the slow h-type conductance, see ref. [28]) were incorporated into the passive leak conductance, which also had a constant density throughout the cell.

To define a parameter set for our model MSO neuron, we used published anatomical data [27] to set the morphology parameters and intracellular recording data [25] to fit the membrane parameters. Basing our morphology parameters on the data from older animals in [27], the single compartment soma had a surface area of 1256 μm^2^ (equaling the surface area of, e.g., a cylinder of length 25 μm and diameter 16 μm) and the two dendritic branches had a length of 150 μm and a diameter of 2.5 μm. The intracellular recordings involved the injection of excitatory postsynaptic current (EPSC) waveforms into the dendrite and recording the voltage responses both locally and at the soma. Thereby the excitatory postsynaptic potential (EPSP) attenuation from dendrite to soma (Fig 1B) and the EPSP halfwidth (i.e., the width at half-maximal amplitude; Fig 1C) were determined. Moreover the experiments showed the input resistance under control conditions and with the KLT-conductance blocked by DTX (Fig 1D). We varied the leak and KLT-conductance densities over a wide range and simulated the experiments to find parameter combinations that fit the data (Fig 1E) and took the best fit as our default parameter set. This resulted in a leak density of 0.86 mS/cm^2^ and a KLT peak conductance density of 13.6 mS/cm^2^.

### Quantifying performance and energy consumption

**Fig 2:**
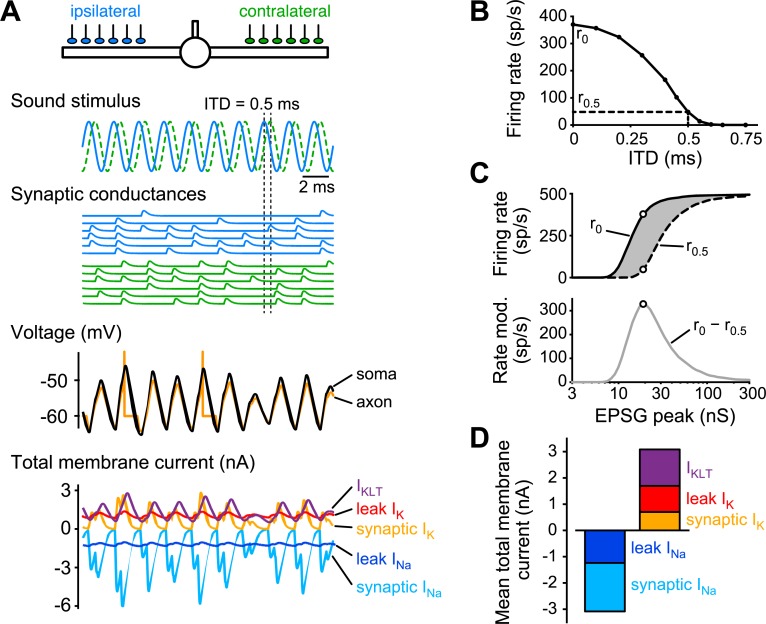
Quantifying MSO cell model performance and energy consumption. (A) Voltage and membrane currents of minimal MSO model in response to 500 Hz pure tone sound wave. Synaptic conductances to the left, ipsilateral (blue) and right, contralateral (green) dendrite are phase-locked to the sound wave, which creates an ITD of 0.5 ms. Somatic (black curve) and axonal (orange) voltages and the total cell membrane currents fluctuate at 500 Hz in response to the synaptic input. Total sodium membrane currents (i.e., summed across entire cell) resulting from leak (dark blue) and synaptic input (light blue) as well as the total potassium currents resulting from *I*_KLT_ (purple), leak (red) and synaptic input (orange) are shown. Model uses default parameters as defined in the Methods. (B) Firing rate of default cell model in response to a 5 s soundwave for a range of ITDs. Maximal firing rate at ITD = 0 ms (r_0_) and firing rate at ITD = 0.5 ms (r_0.5_) are marked. (C) Firing rate at ITD = 0 ms (r_0_, solid black curve) and ITD = 0.5 ms (r_0.5_, dashed curve) are shown when the synaptic peak conductance varies from 3 to 300 nS. Difference between the two curves (gray area in top panel, gray curve r_0_ − r_0.5_ in bottom panel) gives the rate modulation (“Rate mod.”) for the default model as a function of synapse strength. Maximal rate modulation is marked by open circles. (D) Time-averaged total cell membrane currents (summed over entire cell, see bottom panel in A), averaged for a 5 s pure tone sound wave stimulus with ITD = 0 ms.

Having constructed a minimal biophysical model of the MSO cell, we then used it to quantify how its performance, i.e., its ability to encode ITD, relates to the energy it consumes performing this task. We considered one of the simplest auditory stimuli: a pure tone. The anatomical and electrophysiological data used to define the default model represent population averages that were obtained from principal MSO cells with different preferred sound frequencies (note that these frequencies are typically not known in *in vitro* experiments). To account for this fact, we considered a pure tone frequency in the middle of the frequency range that MSO cells, e.g., in gerbils but also humans, respond to (500 Hz). Six separate axonal fibers conveyed the auditory signal to the ipsilateral ear to the ipsilateral dendrite, and, similarly six axonal fibers conveyed the contralateral signal to the contralateral dendrite. The fibers were uniformly distributed across the distal two thirds of each dendrite [29] (Fig 2A, top schematic). The excitatory conductance inputs were phase-locked to the sound wave with a small amount of jitter. Further input noise resulted from the individual fibers occasionally skipping cycles (Fig 2A, “Synaptic conductances”, see Methods). In the example simulation in Fig 2A, the model neuron is encoding the ITD for a sound wave that first arrives at the ipsilateral ear before reaching the contralateral ear 0.5 ms later.

The phase-locked conductance inputs on the dendrites lead to cumulative EPSPs at the soma. For large enough EPSPs the model should generate action potentials. Action potentials in MSO cells are unusually small [30], they are generated in the axon and do not actively backpropagate into the soma and dendrites. We attached an axonal compartment to the soma in an essentially feedforward manner, with a voltage that is strongly coupled to the somatic voltage, but has no influence on the somatic potential. The axonal compartment has a fixed voltage threshold to produce spikes. Hence, when the input fibers are activated synchronously (in this example when ITD = 0, i.e., when the sound source is straight ahead), the EPSPs will summate perfectly and lead to the maximal firing rate (Fig 2B). As the ITD increases, i.e., as the sound source moves towards the ipsi- or contralateral side, the EPSPs will summate less strongly and lead to fewer spikes.

The sensitivity to ITD can be quantified from an ITD-tuning curve in various ways. In order to allow for a systematic exploration of the large parameters space, we quantified the ITD sensitivity as the firing rate difference between ITD = 0 and ITD = 0.5 ms (i.e., r_0_ − r_0.5_), which yielded ~320 spikes/s for the default model. Importantly, the performance (“Rate modulation”) depended entirely on the input strength (Fig 2C). When inputs are too weak, there will be no output, and when the input is too strong, even the out-of-phase inputs (i.e., ITD = 0.5 ms) will saturate the output rate (i.e., 500 spikes/s), leading to zero ITD sensitivity. We therefore needed to ensure that input strength was matched to the cell model. Hence, we determined for each model how it performed as a function of input strength and used the input strength (EPSG peak) that gave it the best performance. For the default model, the EPSG peak per input fiber was ~20 nS, comparable to experimentally obtained estimates [29]. With this EPSG amplitude, four synchronously activated fibers (i.e., two fibers uniformly distributed along each of the two dendrites) were required to generate a spike.

The energy consumption associated with the input processing is largely determined by the costs of the sodium-potassium pump that maintains the sodium and potassium concentration gradients across the membrane, i.e., the costs depend on the sodium and potassium currents during input processing and can hence be determined, to a good approximation, through an “ion-counting” approach (see Methods and ref. [4]). During a simulation, the ionic currents were tracked. The synaptic and leak currents were both considered to consist of a mixture of sodium and potassium currents (see Methods). For the synaptic current, the ratio of sodium to potassium conductance was 2:1, resulting in a synaptic reversal potential of 0 mV. The passive leak current was considered to represent all subthreshold membrane currents other than the synaptic currents and the *I*_KLT_ and had such a ratio of sodium to potassium conductances that the resting potential of the cell was at −60 mV. The total cumulative contributions of sodium and potassium currents across the cell surface from synaptic currents, leak currents and *I*_KLT_ were computed (Fig 2A, bottom panel). Since the pump itself was not explicitly modeled, the total cell sodium and potassium currents (including *I*_KLT_) were exactly balanced at rest (Fig 2D). The energy consumption rate could be computed from the (absolute value of the) mean total cell sodium current, by converting the current into ATP usage rates through the 3:1 ratio of sodium ion extrusion versus ATP consumption. For the default model, we find a mean total sodium current of ~3 nA, which equals 6.2·10^9^ ATPs per second (see Methods) for the ongoing costs associated with input integration for an ongoing 500 Hz pure tone stimulus.

### Saturation of synaptic input currents limits performance and is crucial for energy consumption

We next set out to investigate how the individual intrinsic cell parameters affected performance (i.e., ITD sensitivity) and energy consumption.

**Fig 3:**
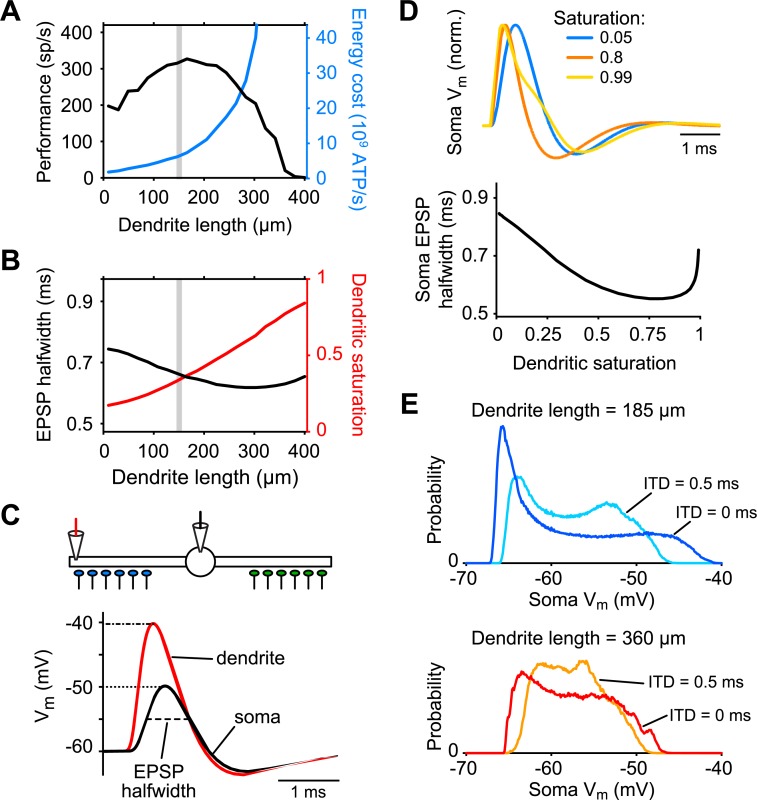
Length of MSO cell dendrites impose a trade-off between performance and energy consumption. (A) Performance (i.e., maximal rate modulation, see Fig 2C; black curve) and energy costs (blue curve) as a function of dendrite length. Stimulus is a 5 s pure tone stimulus of 500 Hz. Default dendrite length is indicated by vertical gray bar. (B) Somatic EPSP halfwidth (black curve) and dendritic saturation (red curve), defined as the peak dendritic voltage normalized by its absolute maximum amplitude (i.e., *E*_rev_−*V*_rest_ = 60 mV). (C) Simulation setup to obtain EPSP halfwidth and dendritic saturation. Voltage is measured in dendrite (red) and soma (black). All synaptic inputs are activated simultaneously with a strength that gives a 10 mV EPSP at the soma (dotted line). Amplitude of the local dendritic response (dash-dotted line) and halfwidth of the somatic EPSP (dashed line) are measured. (D) Somatic EPSP traces are shown for three levels of dendritic saturation (top) and the somatic EPSP halfwidth is shown for the full range of dendritic saturation (bottom). Simulations use the default model; different levels of saturation are achieved by varying synaptic strength, leaving all model parameters constant. (E) Probability distributions of somatic voltage during 5 s simulations with ITD = 0 ms and ITD = 0.5 ms for the default model with a dendritic length of 185 μm (top) or 360 μm (bottom).

We first focus in detail on a morphological parameter: the length of both dendrites. We systematically varied the length of the dendrites from 5 μm up to 400 μm (Fig 3). There was a clear parameter region leading to improved computational performance (quantified by ITD sensitivity, see also ref. [26]). In this region, energy use was low, though not at its theoretical minimum. Specifically, performance of the cell increased with dendritic length up to a maximum around ~320 spikes/s when dendrites were ~150–190 μm long (Fig 3A, black curve). For increasingly longer dendrites, the performance dropped steeply again (see below). In contrast, energy costs increased monotonically with dendrite length (blue curve). The default model parameters representing the experimental data (vertical gray line at 150 μm) were in a range where performance was very good and energy use relatively low.

The dependence of performance and energy costs on dendrite length could be largely explained by two factors: 1) the width of the propagated EPSP at the soma and 2) the amplitude of the synaptic inputs locally in the dendrites (Fig 3B). We quantified both factors by activating all synapses once synchronously with an input strength that depolarized the soma by exactly 10 mV (Fig 3C). First, we turned to functional performance, i.e., ITD sensitivity. We determined the halfwidth of the propagated cumulative EPSP at the soma. This quantity is key to the performance, because the narrower the cumulative EPSP is, the higher the ITD sensitivity (i.e., the rate modulation) can be (Fig 3B, black curve). For the same synchronous stimulus we also determined the amplitude of the response locally in the dendrites. The excitatory conductance inputs have a reversal potential of *E*_rev_ = 0 mV, hence the voltage amplitude will saturate for large inputs, with an absolute maximum amplitude of *E*_rev_−*V*_rest_ = 60 mV. Fig 3B shows the dendritic amplitude normalized by the maximum of 60 mV (red curve). The longer the dendrites were, the stronger the dendritic inputs needed to be to reach the 10 mV depolarization at the soma, i.e., the voltage attenuation from dendrites to soma increased with dendrite length (see Methods) and as a consequence the dendritic saturation increased. Moderate increases in dendritic amplitude helped the performance because more *I*_KLT_ was activated, which narrowed (“sharpened”; see ref. [25]) the EPSPs (Fig 3D, blue versus orange traces). Further increases in dendritic amplitude, however, were detrimental to performance, because the width of the EPSP (yellow trace) increased as it approached saturation. Moreover, saturation led to a decrease of the voltage fluctuations at the soma, hence decreasing the discriminability between ITD = 0 and ITD = 0.5 ms inputs (Fig 3E). Finally, strongly saturated dendritic responses imposed an absolute limit on the input current magnitude and therefore on model parameters, e.g., on the dendrite length: inputs far away from the soma can never generate spikes by themselves, no matter how large the synaptic conductances are, thus leading to zero ITD sensitivity. Taken together, the observed dependence of performance on dendritic length was largely explained by dendritic saturation and changes in somatic EPSP halfwidth.

Next we considered the energy costs. These rise steeply with dendritic length (Fig 3A, blue curve) for two reasons: 1) The dendritic saturation that accompanies longer dendrites naturally correlates with energy consumption; larger synaptic currents boost the synaptic input costs. 2) The total membrane surface area increases with longer dendrites and so do hence the total membrane currents.

### Several cellular properties underlie trade-offs between performance and energy consumption

**Fig 4:**
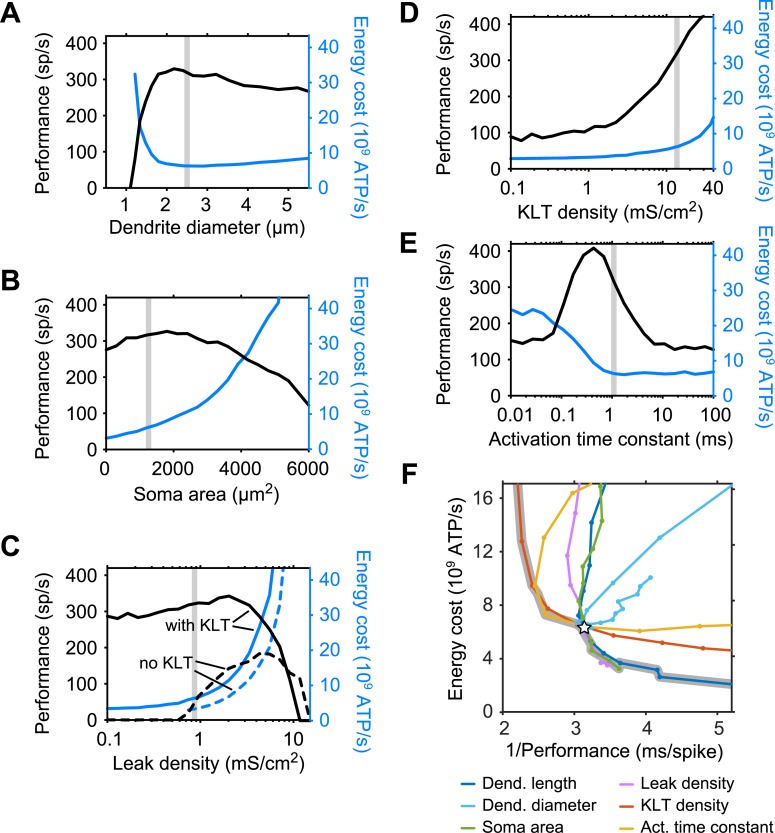
Trade-offs between performance and energy costs when cell morphology and membrane parameters are varied. (A)-(E). Performance (black curves) and energy costs (blue curves) as a function of dendrite diameter (A), soma surface area (B), leak density (C), KLT density (D) and KLT activation time constant (E). Default model parameters are indicated by vertical gray bars. Stimulus is a 5 s pure tone stimulus of 500 Hz. (F) Data from panels A-E and Fig 3A plotted in the energy costs versus performance space. Performance is plotted as its reciprocal value, such that the lower, left corner gives the optimal model with high performance and low energy consumption. Default model is depicted by open star, and the pareto boundary that combines all optimal models as a thick gray curve.

We performed a similar analysis for five additional intrinsic cell parameters (Fig 4; see S1 Text). First, varying two more morphological parameters, the dendrite diameter (Fig 4A) and the soma surface area (Fig 4B), showed that, as for dendrite length (see Fig 3A), the experimentally realistic default parameters (gray bars) were very close to maximal performance while energy consumption was relatively low. Note that the non-monotonic behavior of the energy consumption with dendritic diameter is explained by the counteracting effects of synaptic costs decreasing as the cell becomes electrically more compact with diameter, whereas the increase in cell size increases the costs resulting from the intrinsic membrane currents.

Next, varying cell membrane parameters gave similar results. For the cell’s leak conductance density (Fig 4C, solid curves), the default fitted value was again in a good range where performance was close to maximal and energy cost was low. Importantly, removing the KLT-current from the model (Fig 4C, dashed curves) showed that the model with KLT greatly outperformed the passive model: it performed almost twice as well compared to the best performance of the passive model, while consuming less than half the energy. The contribution of the KLT-current was also revealed when increasing the KLT density (Fig 4D) from very low to high densities. The default, experimentally constrained KLT density (see Fig 1) was situated at a level where performance was very good, while the cost increases that result from KLT were still small. Finally, varying the activation time constant of the KLT-current (Fig 4E) showed that its default value was in a very good range with good performance and small associated costs. A more detailed biophysical discussion of these five morphological and electrophysiological parameters is given in S1 Text.

Taken together, the cell parameters brought about trade-offs between performance and energy use, i.e., better performance was often associated with higher costs. These trade-offs are most clearly depicted by plotting costs versus the reciprocal of the performance (Fig 4F). The most energy-efficient models are located in the bottom left of this space, where energy costs are low and the reciprocal of the performance is low. Note that a value of 2 ms/spike is the best any model can do, as the model is then able to distinguish the in-phase and out-of-phase inputs for every cycle of the 500 Hz stimulus (hence, a 2 ms cycle); larger values correspond to poorer performance. The intersection of all six curves denotes the default model (open star). It appeared that the experimentally constrained default model was, locally in the 6-dimensional parameter space, operating at so-called pareto optimality (thick gray curve) [31], which means that no change in any single parameter could improve performance and at the same time maintain (or lower) the costs. Importantly, along the pareto optimal boundary, performance could be increased through an increase of the KLT density or a decrease of the KLT activation time constant, both manipulations increasing the costs. Conversely, energy consumption was decreased by reducing cell size (dendritic length or soma area) or decreasing the leak conductance, with a consequent decrease in performance.

All above results were obtained by varying a single parameter at a time, while keeping the others fixed. The pareto optimal boundary, however, could possibly be shifted towards lower costs (i.e., downwards) or towards better performance (i.e., leftwards) by combined changes in parameters. We therefore next turned to selected, physiologically relevant trajectories in parameter space.

#### Variation of KLT and leak conductance densities while keeping a stable resting potential

**Fig 5:**
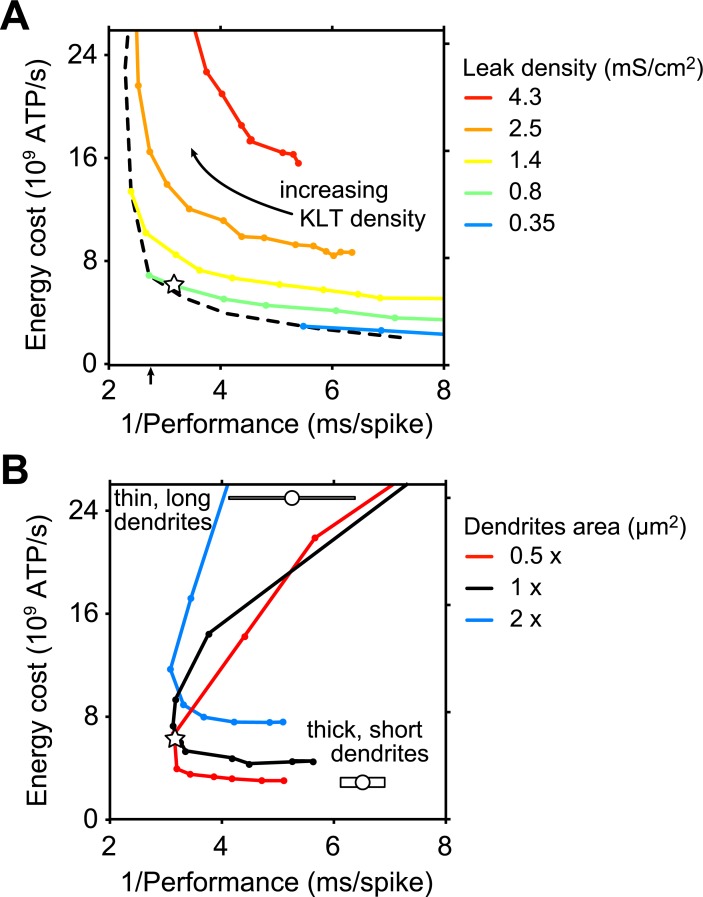
Membrane channel densities allow an improved performance and cell size allows for energy reduction. (A) Energy cost is plotted against reciprocal performance for the default model for parameter combinations where leak density is varied from 0.2 to 10 mS/cm^2^ and KLT density from 0.2 to 40 mS/cm^2^. Dashed curve indicates best models (i.e., best performance for a given energy consumption level) that maintain a resting potential at −60 mV (see Methods). Five curves depict models with a fixed leak density (0.35, 0.8, 1.4, 2.5, 4.3 mS/cm^2^) and range of KLT densities. Default model is depicted by open star. Arrow on abscissa indicates performance of a point neuron model with leak-KLT combination that gives it the best performance. Simulations consist of responses to 5 s long pure tones of 500 Hz. (B) Energy cost is plotted against reciprocal performance for combinations of dendrite diameter and dendrite length that give a constant dendrite surface area. Surface area is equal to the control (black curve; see Methods), twice the control (blue curve) or half the control (red curve). Models with short, thick dendrites are at the bottom right, and models with long, thin dendrites are at the top of the energy costs-performance space. Default model is depicted by open star. Simulations consist of responses to 5 s long pure tones of 500 Hz.

First, we considered the KLT and leak conductance densities together, as KLT was key to improve performance. KLT density cannot be modulated entirely independently from the leak conductance if a neuron needs to maintain a resting potential that is not very far from the firing threshold (at about −50 mV). We hence explored a range of combinations of KLT and leak densities that could maintain a stable resting potential at −60 mV through adjustments of the leak reversal potential (Fig 5A; see Methods). Larger leak and KLT densities allowed for increased performance, shifting the trade-off curve leftwards, but also led to higher costs, shifting the curve upwards. The optimal combinations of KLT and leak densities formed a pareto optimal boundary (dashed curve) [31], highlighting the central trade-off between performance and energy consumption. The fitted model (open star) was located very close to this boundary. The trade-off curves reached a maximum performance of ~440 spikes/s in the depicted energy-performance space.

#### Variation of dendrite length and diameter while keeping a constant cell surface area

Next, we focused on cell size, since decreasing cell size seemed efficient in reducing the energy consumption. In theory, arbitrarily low costs can be obtained when considering a point neuron model with vanishing membrane surface area. Simulations with such a point neuron model yielded a performance of ~360 spikes/s when leak and KLT conductance densities were both optimized to give the maximal performance (Fig 5A, arrow below performance axis). This performance compared well to the ~440 spikes/s performance of a neuron with default dendrite morphology that had both leak and KLT conductance densities optimized for performance (Fig 5A). Hence, the point neuron would be the most cost-efficient MSO cell, showing decent performance for very low costs. In reality, however, there are various constraints driving neuronal design towards a certain minimal size, for example, to create surface area for synapses to connect to. We varied dendrite length and diameter together such that the total cell surface area remained constant (Fig 5B, black curve). This produced a trade-off curve and showed that the default, fitted model gave close to maximal performance with relatively low costs (open circle). This trade-off curve was indeed shifted up or down as the total surface area was varied, not allowing for improvements in performance, but for similar performance with reduced costs.

## Discussion

In this study we investigated how both computational function and energy consumption constrain cell morphology and membrane properties of principal MSO cells. We developed a minimal model of a principal MSO cell and systematically tested how the cell parameters affected performance and energy use. Interestingly, we found that most morphological and membrane parameters showed an optimal range for performance and that the experimentally constrained model, i.e., the model that uses parameters adopted by gerbil MSO cells [25,27], operated within or very close to this range. In contrast, energy use typically varied monotonically with the model parameters. If a wider range of good values for performance was available, the parameters from the experimentally constrained model tended towards lower energy usage. Hence, our findings suggest MSO cell properties are firstly constrained by function and secondarily, if not compromising function, by a reduction of energetic cost.

The key mechanisms that determined model performance and energy consumption were 1) the saturation of the synaptic conductance input and 2) the temporal resolution of the postsynaptic signals as they reach the soma, which was largely shaped by the voltage-dependent membrane properties determined by the low-threshold activated potassium current I_KLT_. The briefer the voltage response, the better. Increased input amplitude in dendrites recruited more KLT-current, yielding narrower, sharper responses [25]. However, when inputs became even larger, saturation of the synaptic currents led to broader postsynaptic responses and decreased voltage fluctuations at the soma, both factors decreasing the performance and steeply increasing the costs. Hence, the entire parameter regime that required large amplitude dendritic inputs (≳ 40 mV) was undesirable from both a functional and an energetic perspective.

The experimentally constrained MSO model operated very close to the set of all models that were optimal with respect to performance and energy use, the so-called pareto optimal boundary (see Fig 4F, models on thick gray curve). This boundary highlights the tradeoff that exists between performance and energy consumption. It depends on the system, including the environment, whether performance or energy use is the more important constraint. Towards better performance, we found that in particular the subthreshold KLT-current, which is present in various cell types in the auditory brainstem [32,33], played a key role, improving the performance well beyond the capabilities of a passive neuron (see also refs. [34,35]). The current has the potential to further improve the performance, but at a substantial energy cost. In the direction of lower costs: cells having short dendrites and small soma surface area are energetically cheaper [36], while they did not lose much on performance. Hence, this indicates further constraints are at play for a neuron to have a certain minimal size, e.g., to create surface area for synapses to connect to [2,3].

### MSO cells are exceptionally suited to study function and energy consumption

MSO cells are particularly well suited to analyze computational and energetic constraints on neural properties, since principal MSO cells are one of the very few cell types in the brain for which the computational function is well characterized. Together with the principal cells from the lateral superior olivary nucleus, the MSO cells are the first in the nervous system to combine input from both ears, allowing for the comparison of arrival times of sound waves at the two ears [21,22]. Furthermore, the highest rates of glucose utilization in the brain are in the structures involved in auditory functions [19]. Firing rates are very high in the early part of the auditory system since activity is triggered directly by sound waves at frequencies of hundreds of hertz, and moreover, spontaneous activity of the auditory nerve (up to ~100 spikes/s) leads to considerable activity even in the absence of sound stimuli [37]. Hence, the high activity levels contribute to high energy costs of the early auditory system. Importantly, the membrane properties enabling such tight tracking of synaptic input are very costly (see below). The increased use of energy of MSO cells because of their computational function was recently supported by Trattner and colleagues [20]. Through immunohistochemistry they quantified metabolic markers for energy consumption and production, indeed showing that MSO cell energy use goes hand in hand with the maturation and refinement of the cellular properties after hearing onset (see also ref. [38]).

### Costs of action potentials

In the present study we focused on the costs resulting from synaptic input processing and did not consider the costs associated with the output spikes. Our arguments for this are, first, that the spike output costs are considerably lower than the input processing costs (see below). Furthermore, the firing rates in the early auditory system are directly related to the sound wave frequencies, hence, optimizing cell properties by reducing costs from output activity would require alternative coding schemes using lower output rates, which is outside the scope of the present study. MSO cells show some properties that are beneficial for reducing the costs resulting from output spikes. In the soma of MSO cells, the action potentials are particularly small [22,29,39] since they are generated in the axon and do not actively propagate back into soma and dendrites, and because the soma imposes a large current sink for the axon [40]. Not only is there no apparent use for such backpropagation—synaptic inputs to MSO cells are not known to show any plasticity, which could rely on such backpropagated signals—but it could potentially even interfere with the extremely precise coincidence detection. This electrical segregation of soma and dendrites from the axon reduces the energy costs related to the action potential output. A further important factor for the output costs is the myelination of axons. The axons from principal MSO cells are indeed myelinated, which first of all speeds up the action potential propagation, but it also has consequences for the costs because the spiking currents are concentrated in the nodes of Ranvier, which are separated from each other by internodes that have a very low capacitance [40,41]. Harris and Attwell [41] computed in detail the costs of action potentials traveling along myelinated fibers from the rat optic nerve. They calculated the costs to be 3.2 × 10^6^ ATPs per spike traveling along a single myelinated fiber of 5.5 mm length. The myelinated axon from a principal MSO neuron projecting to the inferior colliculus would have a similar length in a small rodent (not considering any branching of the axon). Further relevant MSO axon parameters, such as diameter, number of myelin wrappings, internode length, and the overlap of the sodium and potassium currents that underlie the action potential, can vary from a rat optic fiber [40,42], but when we consider the optic fiber as a reference, this would mean that an MSO cell spends 3.2 × 10^8^ to 9.6 × 10^8^ ATPs per second on output spikes (assuming output rates of 100 to 300 spikes/s). Our models show that the costs from synaptic and intrinsic membrane currents during input processing were of similar magnitude (see Fig 2D) and summed up to a consumption rate of 6.2 × 10^9^ ATPs per second per neuron. Hence, the axonal output signaling costs for MSO cells were an order of magnitude lower than the input processing costs.

Note that Harris and Attwell [41] also showed that the myelination of axons is not necessarily energetically advantageous, because of further involved costs that are not directly resulting from the electrical signaling. These additional costs are associated with the myelination process itself and the maintenance of the resting potential of the oligodendrocytes. In the end, the net effect on the total costs of a myelinated axon depends on the axonal firing rates. Because of the typically high firing rates of auditory brainstem cells, myelination is very likely to accomplish significant savings on energy consumption for these neurons.

### Comparison with other cell types

To enable the extremely precise coincidence detection, the MSO cells require very large intrinsic membrane currents. Our default model (Fig 2) showed that during input processing the synaptic costs were of similar magnitude as the intrinsic membrane currents, totaling to a consumption rate of 6.2 × 10^9^ ATPs per second per neuron. How do these costs compare to other neurons? Attwell and Laughlin [4] estimated the energy consumption for a cortical pyramidal neuron to be 0.34 × 10^9^ ATP/s for the resting membrane currents and 1.1 × 10^9^ ATP/s for postsynaptic currents, assuming the synaptic inputs are activated at 4 events/s. Hence, the total input processing costs (1.44 × 10^9^ ATP/s) are only ~23% of that of an MSO neuron, and particularly noticeable are the much lower costs (~10%) of pyramidal neuron subthreshold membrane currents compared to MSO cells. Note though, that a detailed analysis of the costs of input processing in a morphologically reconstructed pyramidal neuron is likely to show higher energy costs, particularly if also the costs of active dendritic processes are considered (see, for example, ref. [43]). Similar estimates of energy consumption have been made for cerebellar neurons [7,8]. Total input processing costs arising from synaptic and intrinsic membrane currents varied from ~0.12 × 10^9^ ATP/s for the small granule cells up to ~3.7 × 10^9^ ATP/s for the larger Purkinje and Golgi cells. Hence, MSO cells are likely among the most energetically expensive neurons in the brain when focusing on the input processing costs. And this is unlikely to change when considering in addition the costs of the output action potentials, since MSO cells fire at very high rates compared to most neurons in the mammalian nervous system. When considering energy density of the tissue, as suggested by Sterling and Laughlin [44], consumption per volume is also high, because MSO cells are small: assuming a volume of 3600 μm^3^ for our minimal model with simplified morphology yields an energy density of 1.7 × 10^6^ ATPs per μm^3^ per second.

Our findings show that for MSO cells the ratio of functional performance and metabolic cost need not always be at its maximum value, contrasting findings in other systems like the retino-thalamic synapses in the visual pathway [45]. Being close to the pareto boundary, however, does not generally exclude a maximization of the efficiency ratio between functional performance and cost; it is well possible, that a system that operates at the maximal efficiency ratio can also be located close to the pareto boundary. Differences in energy efficiency between cell types may, on the other hand, reflect differences in the functional mechanisms and constraints of information transfer as well as the availability of the associated metabolic energy in the system.

Finally, also note that the specialized properties of MSO cells to process high frequency input lead to a high level of energy consumption even in the absence of sound stimuli, in part because of the considerable spontaneous activity levels of the excitatory inputs to MSO cells (~55 spikes/s) [46]. We computed the energy consumption in the absence of relevant sound stimuli to be ~50% of the costs during sound processing.

### Further contributions to energy costs

We focused in this study on the energy costs of the processing of synaptic input. These costs consist of a synaptic contribution and a membrane current contribution (i.e., leak and KLT current). In our calculations we have not included the energy costs of neurotransmitter recycling and vesicle release, which might contribute on the order of 10–20% of total input processing costs [4,8].

A further factor contributing to input processing costs is inhibition. Principal MSO neurons receive glycinergic inhibitory input at the soma [47]. However, the role of inhibition, including its timing and temporal acuity in MSO cells is currently debated [48–50]. The inhibitory input might be involved with shifting the ITD tuning curve [48] or with stabilizing the coincidence detection during ongoing high-frequency input trains [49]. Since the temporally precise coincidence detection performed by MSO cells is possible without inhibition, we therefore did not include inhibition in the current study. The additional costs resulting from inhibitory currents can be expected to scale with the level of excitatory input and with the somatic input conductance, when assuming that the strength of the somatic inhibition keeps pace with the somatic input conductance. Hence, the inhibition costs can be expected to covary with the leak and KLT densities and soma size, and to a lesser extent with dendritic diameter and length.

Importantly, the input processing costs will also depend on the stimulus itself. Because the anatomical and physiological data that we used to constrain our biophysical model were taken from MSO cells with unknown preferred frequencies, we considered a pure tone stimulus with a typical mid-range frequency of 500 Hz. Note that experimental work has not shown a systematic variation of the fast integrative properties [51] along the putative tonotopic axis within the MSO, neither is there evidence of a gradient in morphological properties of the principal cells as has been found in the analogue of the MSO in chicken: the nucleus laminaris [52]. The density of the slow inward rectifying cationic h-type current [28], however, has been shown to be increased for high-frequency neurons in the gerbil MSO. The higher h-current density was hypothesized to counteract temporal summation of inhibitory input in the high-frequency neurons [53]. Increasing sound input frequencies would naturally increase the input integration costs per second. It will be interesting to explore how natural stimuli affect the ongoing costs by MSO neurons, which could be readily explored using available models of the auditory periphery [51,54].

Taken together, our analysis reveals that principal MSO neurons are operating in a functionally very desirable range of their intrinsic parameters, where ITD sensitivity is near-optimal. While energy consumption is known to be very high in these cells, we find that this is not an indication of wasteful processing, but on the contrary, energy consumption is reduced wherever possible without sacrifices to functionality. Our study suggests MSO cells as a prime example for an evolutionarily optimized neuronal design that assigns an important role to energy efficiency, but prioritizes function.

## Methods

### Biophysical model of a principal MSO neuron

A default minimal model of a principal MSO neuron was constructed with biophysical parameters based on experimental data obtained from Mongolian gerbils (*Meriones unguiculatus*) [25,27,29]. The model consisted of a soma compartment and two cylindrical dendritic branches as has been used previously to model MSO cells and cells from the avian analogue of the MSO: the nucleus laminaris [25,26,55]. The default morphology parameters adopted values from anatomical data [27]: the single compartment soma had a surface area of 1256 μm^2^ (e.g.., this equals the surface area of a cylinder of length 25 μm and diameter 16 μm) and the two dendritic branches had length 150 μm and constant diameter 2.5 μm, agreeing with the almost uniform dendrite diameter observed in gerbil MSO dendrites [27]. Note that this morphology is almost identical to the morphology of the model used in [25], except that the dendritic diameter is decreased, in accordance with [27].

The dendritic cables were discretized into compartments of length 0.02 times the passive space constant. The voltage of each compartment *i*, including the soma, evolved according to

$$C_{m}\frac{dV_{i}}{dt}=-g_{L}(V_{i}-E_{L})-{\bar{g}}_{\mathrm{KLT}}w_{i}^{4}z_{\infty }(V_{i}-E_{K})+I_{\mathrm{syn}}(t)+I_{c,i}(t)$$

where the membrane parameters were uniform throughout soma and dendrites with specific membrane capacitance *C*_m_ = 1 μF/cm^2^ and the default densities of the leak conductance and the voltage-dependent low-threshold potassium-current (*I*_KLT_) were *g*_L_ = 0.86 mS/cm^2^ and ${\bar{g}}_{\mathrm{KLT}}$ = 13.6 mS/cm^2^, respectively. Synaptic current *I*_syn_(*t*) was applied to specific compartments (see below). The axial coupling currents *I*_c,*i*_ that a compartment receives from its neighboring compartments were determined by the voltage differences with the neighboring compartments, the axial resistivity *R*_a_ = 200 Ω cm, and the geometry (see, e.g., ref. [56]). The potassium reversal potential was *E*_K_ = −106 mV and the leak reversal potential was set to *E*_L_ = −47.4 mV such that the model had a resting potential of −60 mV. The previously published model of the KLT-current [25] includes a fast activation gate *w* (with an activation time constant at rest of about 1 ms) and a slow inactivation gate *z* which we fixed to its value at the resting potential. Note that the slow inward rectifying cationic h-type current that is present in MSO cells [28] was not modeled explicitly but was considered part of the leak conductance. With the above default biophysical properties the dendrites had a passive DC space constant of 191 μm (excluding *I*_KLT_) and 100 μm for a small voltage deviation from the resting potential when including the voltage-dependent KLT conductance.

In order to fit the membrane conductance densities *g*_L_ and ${\bar{g}}_{\mathrm{KLT}}$ to published electrophysiological data [25] (see Fig 1), we determined the somatic input resistance of the model (under control conditions and with *I*_KLT_ blocked) and the somatic and dendritic voltage response to a dendritic EPSC input where the current waveform was an alpha function with time constant 0.2 ms. As in Mathews et al. [25], we adjusted the amplitude of the EPSC current to obtain a somatic voltage response with an amplitude of 10 mV. We then determined 1) the attenuation of the EPSP amplitude from the dendrite to the soma and 2) the width of the somatic EPSP at half-maximal amplitude (i.e., the EPSP halfwidth). The mean sum squared error for the model’s input resistance (control and *I*_KLT_ blocked, respectively 11.4 MΩ and 36.2 MΩ in ref. [25]), attenuation, and EPSP halfwidth were normalized by the respective means of the 4 variables and then summed up to obtain the total fit error. We chose the model with the smallest error as the default model for our simulations yielding the above default values.

### Numerical simulations to quantify ITD sensitivity

Six separate excitatory input fibers projected to each dendritic branch [29] with each fiber making one synaptic contact. The synapses were distributed uniformly over the distal 2/3 of the dendrites. Synaptic input currents resulted from synaptic conductances *g*_syn_(*t*) that evolved according to an alpha function with time constant 0.2 ms and with a synaptic reversal potential of 0 mV. Peak amplitudes of the synaptic conductances were varied to obtain the maximal ITD sensitivity (see Fig 2C). Synaptic input mimicked the activity that MSO cells receive in response to a 500 Hz pure tone sound wave as in ref. [57]: synaptic inputs were phase-locked to the sound wave and noise was introduced through jitter of input activation times and failures of synaptic activation. Jitter resulted from using a gaussian distribution of synaptic activation times around the peak of the sinusoidal stimulus producing a vector strength of 0.988 (see also [21]). Synaptic failures resulted from forcing each input fiber to have an average firing rate of 240 spikes/s causing them to randomly skip cycles.

Spiking output was computed by voltage threshold crossings in an axonal compartment that was strongly coupled to the soma without affecting the somatic voltage. The voltage threshold was set to −50 mV. Crossing of the voltage threshold was followed by a reset to the resting potential of −60 mV and an absolute refractory period of 1 ms. The passive axonal compartment had a membrane time constant of 0.2 ms and the tight tracking of the soma voltage was achieved by using a coupling time constant of 0.05 ms.

To compute the response of a model to input with an ITD of either 0 ms or 0.5 ms a simulation of 5000 ms was performed using a time step of 0.01 ms.

### Computing energy consumption

We estimated the signaling related energy consumption of each model using the ion counting approach [4]. For this we needed to compute the total amount of sodium ions entering the cell during a simulation. Note that because we did not explicitly model the sodium-potassium pump (which pumps sodium ions and potassium ions in the ratio 3:2, respectively), the number of potassium ions leaving the cell equals the number of sodium ions entering it during a simulation (see Fig 2D). In our biophysical models the sodium ions enter the cell through the leak and the synaptic conductances. We therefore split the leak and synaptic currents into their separate sodium and potassium components. For the leak current this results in:

$$I_{L}(t)=-g_{L,\mathrm{Na}}(t)(V-E_{\mathrm{Na}})-g_{L,K}(t)(V-E_{K})$$

where the leak sodium conductance *g*_L,Na_ and leak potassium conductance *g*_L,K_ can be computed from *g*_L_ = *g*_L,Na_ + *g*_L,K_ and *E*_L_ = (*g*_L,Na_ *E*_Na_ + *g*_L,K_ *E*_K_)/*g*_L_. Note that the balance between these two conductances varies between the different models, because we adjusted the leak reversal potential *E*_L_ such that each model had a resting potential of −60 mV. For the synaptic currents we have:

$$I_{\mathrm{syn}}(t)=-g_{\mathrm{syn},\mathrm{Na}}(t)(V-E_{\mathrm{Na}})-g_{\mathrm{syn},K}(t)(V-E_{K})$$

where the sodium and potassium conductances are computed using *g*_syn_(*t*) = *g*_syn,Na_(*t*) + *g*_syn,K_(*t*) and *E*_syn_ = (*g*_syn,Na_(*t*) *E*_Na_ + *g*_syn,K_(*t*) *E*_K_)/*g*_syn_(*t*). Because the synaptic reversal potential is 0 mV and we consider the reversal potentials *E*_Na_ = 53 mV and *E*_K_ = −106 mV, the ratio of *g*_syn,Na_(*t*) to *g*_syn,K_(*t*) is always 2:1, whereas the absolute values depend on the synaptic peak conductances used.

Over the duration of a simulation we summed the sodium influx into the entire cell to compute the total mean sodium current. For the default model (see Fig 2) we found a total mean sodium current into the cell of ~3 nA. This can be converted into ATP consumption rates by converting current (coulombs per second) into the number of elementary charges per second with the factor 6.242·10^18^ and dividing by three since one ATP is consumed to extrude three sodium ions from the cell. Thus, a 3 nA sodium current results in the consumption of ⅓·3·10^−9^·6.242·10^18^ = 6.2·10^9^ ATPs per second in order to maintain the sodium ion concentration gradient. Note that alternative methods for computing signaling related energy consumption rates are available [58,59] and are expected to lead to similar estimates.

The model was programmed in c-code and used the Crank-Nicolson integration scheme. Results were analyzed using Matlab (The Mathworks, Inc.). The simulation code is publicly available in the ModelDB database, accession number 245424 (http://modeldb.yale.edu/245424).

## Supporting information

S1 Text Detailed analysis of the morphology and membrane parameters on the cell performance and energy use shown in Fig 4A–4E. (DOCX)
