# Supplementary material for: Function and energy consumption constrain neuronal biophysics in a canonical computation: Coincidence detection
Source: PLoS Comput Biol. 2018 Dec 6;14(12):e1006612. doi: 10.1371/journal.pcbi.1006612 (PMC6312336; doi:10.1371/journal.pcbi.1006612)
Supplement: S1 Text — (DOCX) [file pcbi.1006612.s001.docx]

# **S1 Text - Detailed analysis of the morphology and membrane parameters on the cell performance and energy use shown in Fig 4A-E**

**Dendrite diameter.** We varied the dendrite diameter of the model from 0.5 μm to 5.5 μm (Fig 4A). Just as the dendrite length, this parameter also had a major role in determining performance and energy consumption. Importantly, for very thin dendrites (< 1 μm), the neuron could not reach firing threshold, because even a maximally depolarized thin dendrite was not able to deliver enough current to charge the soma (i.e., a large impedance mismatch, in addition to electrically longer dendrites). When the diameter exceeded 1 μm, the ITD sensitivity increased steeply up until a maximum at ~2 μm (black curve). At the same time the costs for input integration dropped steeply (blue curve). As for dendritic length (see Fig 3A), the experimentally realistic default dendrite diameter of 2.5 μm (vertical gray line) was very close to maximal performance and minimal energy consumption. Note that the non-monotonic behavior of the energy consumption is explained by the counteracting effects of synaptic costs decreasing as the cell becomes electrically more compact with diameter, whereas the increase in cell size increases the costs resulting from the intrinsic membrane currents.

**Soma surface area.** The third and last morphology parameter, the soma surface area, showed similar profiles for performance and energy use (Fig 4B) as the dendrite length (see Fig 3A), though the dependence was weaker, especially within a range around the default value (~1260 μm^2^). Results can be understood from the larger soma requiring stronger inputs for it to reach a 10 mV depolarization, eventually leading to saturation of the input (at ~6000 μm^2^). The default model was in a very good range where performance was good and energy costs were relatively low.

**Leak conductance density.** Turning to the first of three membrane parameters, we systematically varied the leak conductance density from 0.1 to 15 mS/cm^2^ (Fig 4C). We found a modest, steady increase in performance until about 2–3 mS/cm^2^ after which performance dropped steeply (solid black curve). An increasing leak density was accompanied by an increase in costs (solid blue curve). The default fitted value of ~1 mS/cm^2^ was again in a good range where performance was close to maximal and energy costs were relatively low. A striking result was the good performance that could be achieved with very low leak densities. The reason for this is clear when removing the other membrane conductance: KLT (Fig 4C, dashed curves). This led to a very strong drop in performance; only a relatively limited range of leak densities showed any ITD sensitivity (between 1–15 mS/cm^2^). Over practically the entire range, the model with KLT greatly outperformed the passive model, highlighting the central role of KLT conductances in MSO principal cells (and many other auditory brainstem neurons). Importantly, the default, experimentally constrained model with KLT (vertical gray bar) performed almost twice as well compared to the best performance of the passive model, while consuming less than half the energy.

**KLT conductance density.** We subsequently varied the density of the KLT conductance from 0.1 to 40 mS/cm^2^ (Fig 4D). For low densities, the model performed close to a passive model (see Fig 4C, dashed curves). With increasing KLT density the performance increased monotonically. One limiting factor was that as the potassium conductance density became larger, it would by itself hyperpolarize the resting potential further and further. Here, we maintained the resting potential by considering that the other membrane conductances (summarized in the passive leak conductance) have a combined reversal potential that counteracts the hyperpolarization. When the KLT density became ~22 mS/cm^2^, the stable resting potential was only achievable with a very depolarized leak reversal potential of −40 mV, which is equal to the reversal potential of another (slow) voltage-dependent current in MSO cells: the h-type conductance [28]. Hence, such large KLT densities would require that the leak is made up entirely of the h-current, which we therefore considered as a natural limit to the KLT density. The performance increase with KLT density is explained by its strong sharpening effect on the EPSP [25]. Only for very high KLT densities did KLT attenuate the EPSP amplitude such that significantly stronger inputs were required, thereby increasing the costs. The default, experimentally constrained KLT density (see Fig 1) was situated at a level where performance was very good, while the cost increases resulting from KLT were still small.

**KLT activation time constant.** The final model parameter that we considered was the activation time constant of the KLT current. This voltage-dependent parameter has been measured in experiments to be around 1 ms at the resting potential (and slightly faster at more depolarized potentials) [25]. Interestingly, the parameter proved to have a strong effect on performance and costs of input integration (Fig 4E). For large activation time constants (>10 ms), the dynamics of the KLT current were effectively ‘frozen’ and the neuron acted as a passive neuron. As the activation time constant decreased below ~10 ms the performance improved, showing a very pronounced peak for values around 0.5 ms, which gave a rate modulation of ~400 spikes/s. For an even faster KLT current, down to 0.1 ms, the performance dropped again. In contrast, the costs showed a monotonic dependence: the faster the current, the more energy was spent. The default parameter (~1 ms) was in a very good range with good performance and small associated costs. The effects on performance and costs can be understood from the sharpening effect of KLT in the following way. The KLT-current activates upon depolarization. For an appropriate activation time constant, the hyperpolarizing KLT current gets mostly activated during the falling phase of the EPSP, creating very narrow voltage transients. When KLT is faster, it already activates during the rising phase of the EPSP, counteracting the depolarization without significantly sharpening the EPSPs. As a consequence, the costs grow because input strength has to increase to generate spikes.
